# CT scanning technology to asses the genetic and phenotypic correlations, and selection potential of carcass traits in mutton sheep

**DOI:** 10.5713/ab.250491

**Published:** 2025-11-25

**Authors:** Yuan Zhao, Guoxing Jia, Xiaoxue Zhang, Huibin Tian, Deyin Zhang, Yukun Zhang, Xiaolong Li, Jiangbo Cheng, Liming Zhao, Quanzhong Xu, Xiaobin Yang, Zongwu Ma, Dan Xu, Fadi Li, Weimin Wang

**Affiliations:** 1 State Key Laboratory of Herbage Improvement and Grassland Agro-Ecosystems, Key Laboratory of Grassland Livestock Industry Innovation, Ministry of Agriculture and Rural Affairs, Engineering Research Center of Grassland Industry, Ministry of Education, College of Pastoral Agriculture Science and Technology, Lanzhou University, Lanzhou, China; 2 College of Animal Science and Technology, Gansu Agricultural University, Lanzhou, China

**Keywords:** Carcass Traits, Genetic Parameters, Genomic Selection, Sheep

## Abstract

**Objective:**

This study aims to evaluate the variations in carcass fat percentage (CFP), carcass muscle percentage (CMP), carcass bone percentage (CBP), meat-to-bone ratio, carcass weight, loin eye area (REA), backfat thickness (BF), the total tissue depth of muscle and fat at the twelfth rib, 110 mm from the midline (GR), and dressing percentage in 574 mutton sheep using CT scanning technology. It also seeks to estimate the genetic and phenotypic correlations, as well as estimated genomic selection accuracy provides reference for breeding of mutton sheep carcasses.

**Methods:**

Phenotypic data from National Mutton Sheep Testing Station; uniform rearing for 155 days. CT scans were used to obtain body images of the sheep, and CT-Calc2012 software was employed to trait determination. Genomic data were sequenced using second-generation sequencing, and SNP calling was performed with GATK. A mixed linear model incorporating both genomic and pedigree data was used to estimate genetic parameters for various traits. Cross-validation through ten-fold was conducted to assess genomic selection accuracy, and both direct and correlated selection responses for carcass traits were analyzed.

**Results:**

The coefficient of variation for each trait ranging from 8.86% to 25.12%. All carcass composition traits demonstrated medium to high heritability (0.38–0.51). A strong genetic correlation was found between BF, REA, CFP, and CMP. The genomic selection accuracy for carcass traits ranged from 0.29 to 0.43, suggesting potential for genomic selection in mutton sheep breeding. The genetic progress of CMP and CFP after direct and indirect selection was tested, showing that indirect improvement of CMP and CFP was most significant when selecting for BF and REA.

**Conclusion:**

The study indicates that BF and REA are valuable traits for improving carcass composition in mutton sheep breeding, with genomic selection offering prospects for breeding carcass traits more effectively.

## INTRODUCTION

The efficient production of animal protein for human consumption is a key objective in livestock farming. Lamb meat serves as a vital source of protein in the diets of many countries, where it is used to prepare traditional dishes enjoyed around the world [1]. As awareness of healthy living continues to grow, the demand for low-fat, high-protein lamb products is also increasing. Ensuring the lamb industry produces superior mutton with exceptional quality and enhanced nutritional value is crucial [2]. However, current intensive farming practices, which are designed to improve feeding efficiency, may contribute to a decline in the overall quality of mutton. A primary strategy for achieving superior products is to improve the carcass composition of livestock. The higher the lean meat percentage of the carcass is, the higher the unit price of the carcass will be. In production, the energy required for fat deposition is much higher than that for muscle. Product quality can be enhanced when animals allocate more energy to developing valuable, edible body parts Therefore, one of the breeding goals for mutton carcasses is to increase the lean meat percentage [3].

In response to these challenges, an increasing number of breeders have recognized the need to improve meat quality and have begun focusing on enhancing the carcass muscle content to boost the value of mutton sheep. However, due to the unique growth characteristics and economic value of mutton sheep, improving their carcasses is often more challenging compared to species like pigs or beef cattle. In pig and cattle breeding programs, the muscle and fat content in the carcass is difficult to measure directly [4,5]. However, through genetic research, certain indicative traits have been used for indirect selection to enhance muscle and fat content. The results show a significant negative correlation between backfat thickness (BFT) and muscle content [6], so BFT is typically used as an indicator for assessment. Previous research has also found that the loin muscle area (LMA) is negatively genetically correlated with BFT and positively genetically correlated with lean meat percentage [7]. By focusing on traits such as muscle area, BFT, and lean meat percentage, carcass quality can be improved.

Although the importance of carcass improvement is widely acknowledged in sheep, progress in trait enhancement has been slow due to the challenges in accurately determining muscle and fat content in carcasses. Breeders are currently unable to effectively guide breeding for muscle and fat content in sheep carcasses because of the lack of precisely estimated genetic parameters [8]. While some studies have estimated the genetic parameters of various carcass traits in different sheep breeds [9,10], there are still relatively few research reports on traits that are difficult to measure. As a result, breeding for certain carcass traits in sheep is often guided by findings from studies conducted in other species. It is essential to determine whether LMA, LMD, and BFT significantly influence carcass traits such as leanness, in mutton sheep [11]. Examining the genetic relationships among these traits is key to addressing the research question.

We obtained precise phenotypic data for sheep carcasses using Computed Tomography and established a reference population for large-scale enomic selection of carcass traits in sheep. The objective of this study was to obtain (co)variance components, heritability, genetic correlations, phenotypic correlations and the accuracy of genomic selection for carcass traits, including challenging yet crucial traits such as carcass muscle, fat, and bone percentages, conventional traits like BFT, eye muscle area, Greville, carcass weight (CW), and slaughter rate. Furthermore, the study aimed to predict the direct and correlated responses for carcass composition traits when selection is applied to carcass traits in sheep. This information will facilitate more efficient breeding selection for desirable meat characteristics.

## MATERIALS AND METHODS

### Experimental animals and phenotyping

The National Mutton Sheep Testing Station, which is situated between 38°34′ and 40°15′N latitude and 116°43′ and 118°04′ E longitude, provided the phenotypic data used in this investigation. The slaughter time for fattening mutton sheep is between 5 and 8 months old. To save the time cost of measurement, we chose to conduct the phenotypic measurement at the age of 155 days. After all the sheep were weaned at 90 days of age. After weaning, every sheep was kept in the same testing station and fed the same diet. A combination of silage and added concentrate feed made up the diet. The phenotypic characteristics of each sheep were measured after they were grown for 155 days. Before the measurements, the animals were fasted for 24 h with no feed and water provided. In this experiment, a total of 574 ram individuals were measured, including 283 Australian White sheep, 97 Dorper sheep, and 194 individuals from their hybrid population.

**Figure 1:**
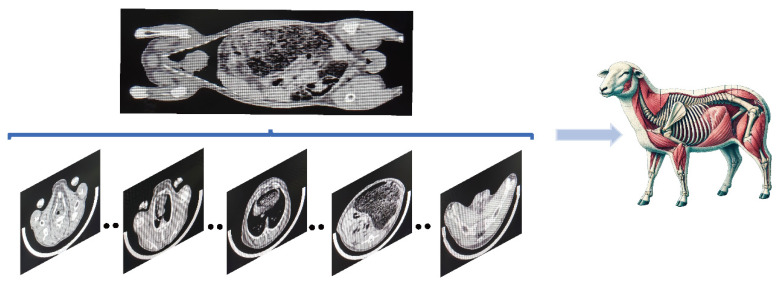
Phenotypic data collection process. The image shows how CT scanning divides the live body into several cross-sectional images, using image recognition and modeling to estimate the weight of muscle, fat, and bone in the carcass.

After anesthetizing the animals, a NeuViz 16 Classic CT scanner was used to scan the entire body starting from the cervical vertebrae. An average of 180 scanned images were generated for each individual, as shown in Figure 1. Using CT-segment, the body and internal cavity images were separated to analyze the fat, muscle, and bone images. The CT-Calc2012 software, along with the built-in sheep model, was used to estimate the carcass fat, muscle, and bone weights from the images. Select the CT images between the 12th and 13th rib intervals, and use ImageJ (USA, 1.53) software to measure the loin eye area (REA), BFT, and GR value traits.

### Genomic data

Whole-genome resequencing was performed on all 574 sheep in the reference population. Genomic DNA was isolated from whole blood of each sample using an EasyPure Blood Genomic DNA Kit (TransGen Biotech) following the manufacturer’s instructions. Agarose gel electrophoresis and the A260/280 ratio were employed to assess DNA purity and integrity. According to the manufacturer’s protocol, 1.5 μg of high-quality genomic DNA was used to create paired-end sequencing libraries with an insert size of approximately 500 bp for each sample. These libraries were then sequenced on the Illumina NovaSeq PE150 platform (Illumina).

The SRAToolkit (v.2.9.2) was used to convert all fastq files from raw sequencing [12] data, and Trimmomatic (v.0.36) was applied to filter them and produce clean reads [13]. The clean reads were then mapped onto the sheep reference genome Oar_rambouillet_v1.0 using BWA (Burrows-Wheeler Aligner) software (v.0.7.8) with default settings [14]. SAMtools (v.1.12) was then used to convert the mapping results into BAM format and to sort them. PCR duplicates were removed using Picard software (v.2.26.2) (https://broadinstitute.github.io/picard/) prior to variant calling. The Bayesian method in the Genome Analysis Toolkit (GATK) v.3.4.0 was used to call SNPs from the BAM data [15]. The VariantFiltration module was employed to filter out excessively heterozygous variants (ExcessHet>54.69) marked with ExcessHet. High-quality SNPs were retained for further analysis using VCFtools (v.0.1.14) based on the following criteria [16]: minor allele frequencies≥5%, minimum coverage depth>3, and missing rates<0.6. After quality screening, all SNPs were further annotated using SnpEff v4.3t based on the gene annotations of the sheep reference genome [17] Oar_rambouillet_v1.0. SNPs were categorized into exonic regions (synonymous or nonsynonymous SNPs), intronic regions, and upstream and downstream regions according to genome annotations.

### Genetic parameters estimated

The pedigree dataset has recorded the parentage of each individual at the National Mutton Sheep Testing Station since 2015. Through multiple generations of breeding, the pedigree information has been meticulously traced. In addition to the individuals in this study, the pedigree data of 63,014 sheep were also included in this study, enabling the construction of an accurate additive genetic relationship matrix among individuals.

To determine the additive genetic variance components for various carcass traits, we used HIBLUP [18] software to fit a mixed linear model. Specifically, variance components for single- and two-trait single-step genomic BLUP analysis were estimated using the restricted maximum likelihood method with average information (AIREML) [19]. To estimate variance components and random effects, we constructed the additive genetic relationship matrix for the sheep using both pedigree data and genomic estimates.


(1) $$H=\left[\begin{array}{cc} A_{11}+A_{11}A_{22}^{-1}\left(G_{w}-A_{22}\right)A_{22}^{-1}A_{21} & A_{12}A_{22}^{-1}G_{W}\\ G_{W}A_{22}^{-1}A_{21} & G_{W} \end{array}\right]$$

Where *A*_11_ and *A*_11_ are the Submatrices of the A matrix for non-genotyped and genotyped individuals, respectively. *A*_12_ and *A*_21_ are submatrices of the A matrix, representing the relationship between genotyped and non-genotyped individuals. *G_w_* = (1−*alpha*)×*G_adj_*+*alpha*×*A*_22_; Here, *G_adj_* is modified using straightforward linear regression using the initial genomic association matrix *G*. The adjustment process is as follows in the following formula.


(2) $$G=\frac{ZZ^{T}}{\text{tr}\left(Z^{T}\right)/n}$$

(3) $$Avg{\left(diag(G)\right)}^{★}a+b=Avg(diag(A_{22}))$$

(4) $$Avg{\left(offdiag(G)\right)}^{★}a+b=Avg(offdiag(A_{22}))$$

(5) $$G_{adj}=G\times a+b$$

In this matrix, Z consists of elements and values equal to (2−2*p_i_*), (1−2*p_i_*), −2*p_i_* corresponding to genotypes AA, Aa and aa, respectively, where pi represents the frequency of allele A at the i.-th SNP.

The analysis of variance components for single trait mod was conducted with an animal model structured as follows:


(6) y=μ+Xβ+Zg+e

Where *y* is the vector of phenotypic values, *β* is the vector of fixed effects, the fixed effects include the year of measurement, the shed of measurement, breed information and the season of measurement, *g* is the vector of direct additive genetic effects, *X* and *Z* are incidence matrices relating *β* and *g* to *y*, and *e* is the vector of residual effects. It was assumed that *E*[*y*] = *Xβ*; Var(*g*) = H⊕*V*; and Var(*e*) = I⊕*Vg*, where *Va* is the (co)variance matrix for the additive genetic effects, *Ve* the covariance matrix for residual effects, *I* is the identity matrix, and ⊕ is the direct Kronecker product.

Additionally, using the mixed bivariate animal models, estimate the genetic and residual correlations between the traits. The mixed bivariate animal models, as follows:


(7) $$\left[\begin{array}{c} y_{1}\\ y_{2} \end{array}\right]=\left[\begin{array}{cc} X_{1} & 0\\ 0 & X_{2} \end{array}\right]\left[\begin{array}{c} b_{1}\\ b_{2} \end{array}\right]+\left[\begin{array}{cc} z_{1} & 0\\ 0 & z_{2} \end{array}\right]\left[\begin{array}{c} g_{1}\\ g_{2} \end{array}\right]+\left[\begin{array}{c} e_{1}\\ e_{2} \end{array}\right]$$

Where: *y*_1_ and *y*_2_ are the vectors of phenotypic observations for the traits 1 and 2; b1 and b2 are the vector of fixed effects (consistent with the single trait model); g1 and g2 are the vectors of additive genetic effects for the two traits; and e1 and e2 are the vectors of random errors; X1 and X2 are the matrices of incidence of n×p order associating each observation (n) to the pertinent level of Computed Tomography month; Z1 and Z2 are the matrices of incidence of n × s order associating each observation (n) to each animal (s).


(8) $$\left[\begin{array}{l} g_{1}\\ g_{2}\\ e_{1}\\ e_{2} \end{array}\right]=\left[\begin{array}{cccc} Hg_{11} & Hg_{12} & 0 & 0\\ Hg_{21} & Hg_{11} & 0 & 0\\ 0 & 0 & Ie_{11} & Ie_{12}\\ 0 & 0 & Ie_{21} & Ie_{22} \end{array}\right]$$

Where: *g*_11_ was the additive genetic variance for the direct effect for trait 1; *g*_12_ was equal to *g*_21_ and was the additive genetic covariance between the two traits; *g*_22_ was the additive genetic variance for direct effect for trait 2; *e*_11_, *e*_12_, *e*_21_and *e*_22_ were the variance and covariance matrices for the residual effect; H was the relationship matrix between all animals.

### The accuracy of genomic selection and cross validation schemes

Ten-fold cross-validation was applied to test the prediction accuracies across different traits. The validation method involved randomly sampling 54 animals as the validation set (rounding for balanced fold sizes), while the remaining animals were used as the reference set of genomic selection in HBLUP, with 10 replicates. The prediction accuracies were estimated using the genetic estimated breeding values and the observed corrected phenotypic values for each trait. The formula for accuracy estimation is as follows:


(9) $$Accuracy=\sqrt{\frac{cov\left((H)EBV_{w},Pheno_{adj}\right)}{\sigma_{a}^{2}}}$$

Where (*H*)*EBV_W_* is the vector of estimated breeding value; the *Pheno_adj_* is the vector of corrected phenotype value; $\sigma_{a}^{2}$ is the additive effect variance components.

### Direct and correlated response to selection

Correlated responses for carcass fat percentage (CFP) or carcass muscle percentage (CMP), considering selection for carcass traits, were obtained under single-trait selection. The expected responses to direct single-trait selection were estimated for all evaluated traits using the following equation [20].


(10) $$\Delta G_{Y}=(r_{tiY}\times i_{Y}\times \sigma_{aY})/GI$$

Where Δ*G_Y_* is the genetic in gain in trait Y per generation; *r_tiY_* is the accuracy of genetic prediction of Y; *i_Y_* is the intensity of selection for trait Y; σ*_aY_* is the standard deviation of the variance of additive effects. *GI* is the generation interval. To calculate the expected response to direct or indirect selection for CFP and CMP, a 1.5years generation interval was assumed. The selection intensity was considered equal to 1.2, corresponding to the selection of 10% and 60% of males and females, respectively.

Correlated responses for CFP and CMP when direct selection was applied for them or for carcass traits were estimated as follows [21]:


(11) $$\Delta G_{Y|X}=(r_{gXY}\times r_{tiX}\times i_{X}\times \sigma_{aY})/GI$$

Where Δ*G_Y_*_|X_ is the genetic gain per generation in trait Y, given selection for X; *r_gXY_* is the genetic correlation between X and Y; *r_tiX_* is the accuracy of the genetic prediction of X; *i_X_* is the intensity of selection for trait X; σ*_aY_* is the standard deviation of the variance of additive effects on trait Y.

The relative efficiency of selection (RES) [22] was calculated as the ratio between the direct and indirect response to the selection, as below:


(12) $$RES=\frac{\Delta G_{Y}}{\Delta G_{X|Y}}\times 100$$

## RESULTS

### Descriptive statistics of carcass and phenotypic correlations

The descriptive statistics of the carcass traits for the mutton sheep are presented in Table 1. Since the units of different traits are inconsistent, in order to compare the differences in the degree of dispersion among different traits, we calculated the coefficients of variation for each trait. Notably, the highest coefficients of variation were observed for BF, GR, and CFP, with traits related to carcass fat exhibit considerable genetic variation within the population. The coefficient of variation for CMP is lower compared to that for CFP. Regarding carcass composition, both the maximum and average values for CMP are higher than those for CFP. This suggests that muscle is the dominant component in the carcass, with smaller variation between individuals, while fat, though secondary, exhibits greater individual differences.

**Figure 2:**
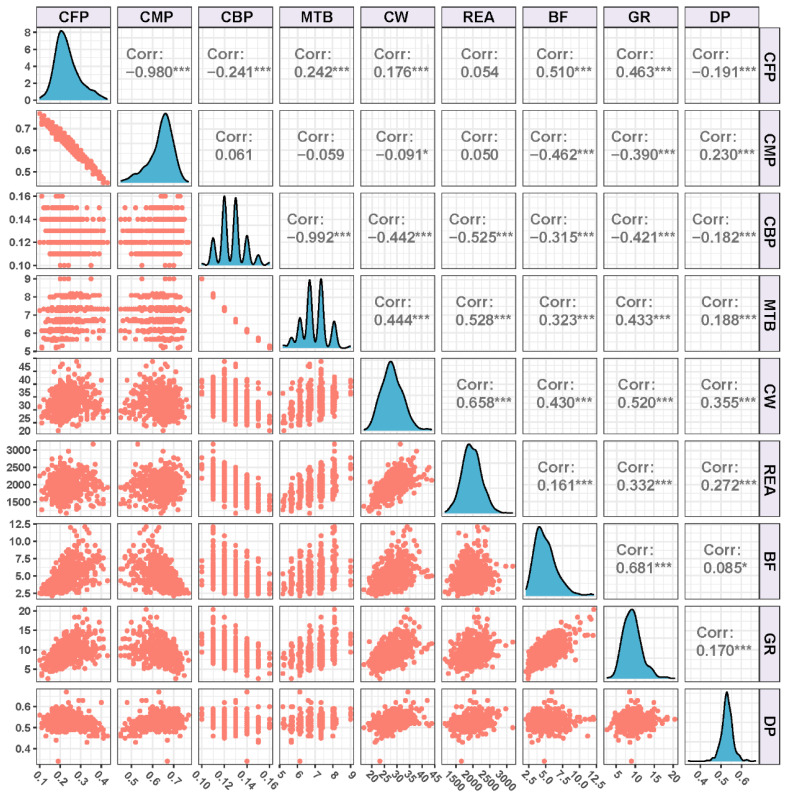
Phenotypic Pearson correlations between carcass traits in mutton sheep. The top right corner of the image shows the Pearson correlation, the diagonal represents the phenotypic probability density distribution, and the bottom left corner displays the linear relationships between pairs of phenotypes. The values on the vertical and horizontal axes represent the range of each trait; * p<0.05, ** p<0.01, *** p<0.001 for significance of correlation. CFP, carcass fat percentage; CMP, carcass muscle percentage; CBP, carcass bone percentage; MTB, meat-to-bone ratio; CW, carcass weight; REA, loin eye area; BF, backfat thickness; DP, dressing percentage.

The phenotypic correlations are shown in Figure 2. CFP is significantly positively correlated with BF and GR, with Pearson correlation coefficients of 0.45 and 0.76, respectively. CMP is also significantly negatively correlated with BF and GR, with Pearson correlation coefficients of −0.39 and lower. From a phenotypic correlation perspective, BF and GR can indirectly reflect the levels of carcass fat and muscle content. Interestingly, the eye muscle area shows no correlation with the proportion of carcass composition. Instead, it has a strong phenotypic correlation with CW and meat-to-bone ratio (MTB), with correlation coefficients of 0.52 and 0.65, respectively. Additionally, the proportion of bone mass in the carcass is strongly phenotypically correlated with CW. Compared to CFP (0.17) and CMP (−0.09), carcass bone percentage (CBP) shows an even stronger phenotypic association (−0.44) with CW.

### Genetic parameters and genetic correlations estimates

Heritability estimates for carcass composition distributions obtained through CT scans are moderate, ranging between 0.38 and 0.51 for different carcass composition (Table 2). The heritability of CMP is 0.47, while the heritability of CFP is 0.51, and that of CBP is 0.39. The heritability of all carcass composition traits is notable. In this study, the image data of the cross-section between the 12th and 13th ribs of live animals was obtained under anesthesia regarding the phenotypes of local cross-sectional slices of the carcass. Compared with the measurement after slaughter and cutting, not only can the measurement position remain consistent, but also more accurate data can be obtained without damaging the loin eye area and fat thickness [23]. Thus, it has a stronger guiding significance compared with the results of traditional measurements. Aside from GR, which has a heritability of 0.15, both REA and BF show moderate to high heritability, with values of 0.37 and 0.50, respectively. Among these traits, DP has the lowest heritability at 0.14, and the heritability of CW has the highest at 0.54.

**Figure 3:**
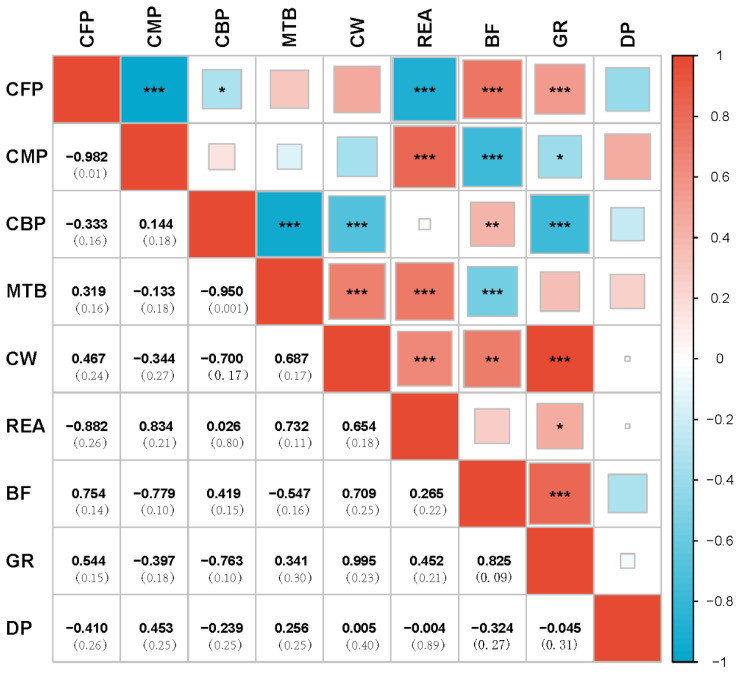
Genetic correlations (±SE) between carcass traits in mutton sheep. The bottom left corner displays the genomic correlation values (±SE), while the top right corner contains the visual heatmap and significance. * p<0.05, ** p<0.01, *** p<0.001 for significance of correlation. CFP, carcass fat percentage; CMP, carcass muscle percentage; CBP, carcass bone percentage; MTB, meat-to-bone ratio; CW, carcass weight; REA, loin eye area; BF, backfat thickness; DP, dressing percentage; SE, standard error.

The estimated genetic correlations are shown in Figure 3. These correlation estimates range from low to high magnitudes, both positive and negative, depending on the carcass traits (−0.98±0.01 to 0.99±0.23). A strong negative genetic correlation is found between CFP and CMP in carcass composition traits (−0.98±0.01). Five correlations between different carcass composition traits (CMP and CFP) and local cross-sectional slices of the carcass (REA, BF, and GR) are above 0.50. For example, CFP shows strongly genetically correlated with REA (−0.88±0.26) and BF (0.75±0.14), while CMP shows similar correlations with REA (0.83±0.21) and BF (−0.77±0.10). MTB also displayed a strong correlation with REA (0.73±0.11) and BF (−0.54±0.16). Overall, a high correlation is observed between REA and BF characteristics and the carcass meat fractions. DP shows weak associations with all other carcass characteristics, with the strongest correlations identified with CMP (0.46±0.25), while the lowest correlations are found with REA (0.04±0.89) and CW (0.05± 0.40). CW is found to be strongly correlated with carcass traits other than DP (−0.34±0.27 to 0.99±0.23), with the highest correlation observed with GR.

### The accuracy of genomic selection

**Figure 4:**
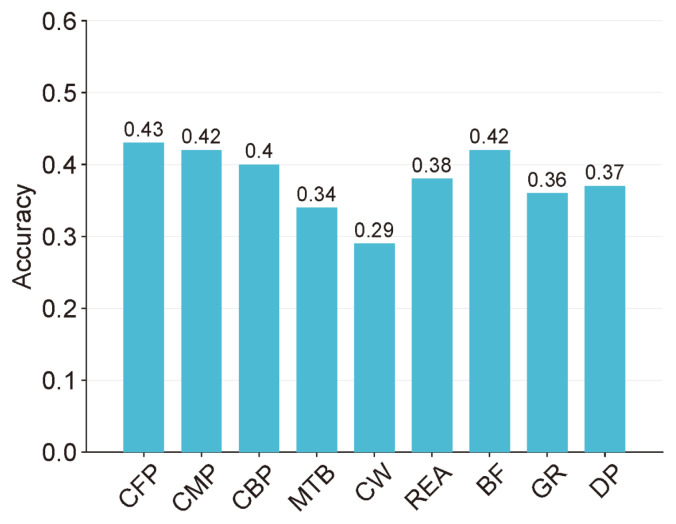
Accuracy of genomic selection using ten-fold cross-validation. The image shows the estimation of genomic selection accuracy for each trait, with the vertical axis representing genomic selection accuracy value. CFP, carcass fat percentage; CMP, carcass muscle percentage; CBP, carcass bone percentage; MTB, meat-to-bone ratio; CW, carcass weight; REA, loin eye area; BF, backfat thickness; DP, dressing percentage.

The genomic selection accuracy shows in Figure 4 is calculated as the average Pearson correlation between the corrected phenotype and the EBV of carcass traits. The accuracy of genomic selection ranges from 0.29 to 0.43, indicating relatively high accuracy. The accuracy for carcass composition traits is the highest, with values of 0.43 (CFP), 0.42 (CMP), and 0.40 (CBP), respectively. The accuracy for traits related to local cross-sectional slices of the carcass follows a similar trend, with values of 0.38 (REA), 0.42 (BF), and 0.36 (GR), respectively. Among these traits, CW had the lowest accuracy at 0.29.

### Response to selection

The response to direct selection for CFP and CMP, as well as indirect selection through carcass traits, is presented in Table 3. The results indicate that the response to direct selection for CFP is greater than the response to indirect selection through feeding carcass traits. The correlated responses for CFP when selecting for CMP (−2.20), CBP (−0.71), MTB (0.58), CW (0.72), REA (−1.79), BF (1.69), GR (1.05), or DP (−0.81) are all lower than the response to direct selection for CFP (2.30). Similarly, direct selection for CMP traits produced higher responses than indirect selection. For both traits, the RES shows that selecting for BF and REA is the most effective in improving CMP (128.30 for REA, 135.74 for BF) and CFP (S132.51 for REA, 128.35 for BF), in addition to direct selection.

## DISCUSSION

This study, for the first time, characterizes the proportions of muscle, fat, and bone within the carcasses of sheep in a large-scale population, and estimates the phenotypic and genetic correlations between these traits and conventional carcass characteristics. A genomic selection reference population was created based on the data, and the accuracy of genomic selection for carcass traits was estimated. Muscle content makes up the largest proportion of carcass composition, with its coefficient of variation being much smaller than that of carcass fat content within the population, and similar to the coefficient of variation of bone proportion. Additionally, the BF trait, which is used to assess carcass fat content in pigs, exhibited a large coefficient of variation in this study and showed a high phenotypic correlation of 0.51 with carcass fat content. Previous research has found that the coefficient of variation for lean meat percentage in pigs is 5.58%, while the coefficient of variation for BF reached 18.54% [24], a trend that aligns with the findings of this study. It was found that the coefficient of variation for MTB was still lower than that for CFP, suggesting that there is significant variation in internal fat deposition within the carcass and considerable potential for selection.

In previous studies, eye muscle area has often been used to assess leanness in swine and beef carcasses. However, this study did not find a significant phenotypic correlation with leanness. Instead, a strong phenotypic correlation was observed between BF and leanness, which is consistent with the findings of Davoli et al [24] However a significant phenotypic correlation was found between eye muscle area and CW. In beef cattle studies, eye muscle area has been positively genetic correlated with individual live weight and body size [25,26]. Larger eye muscle areas were found in heavier pig breeds, and higher muscle fiber densities were observed in the longest muscles along the pig’s back. Muscle fiber density is significantly correlated with the growth and development of carcass muscles [27]. Muscle is the largest composition in the carcass. muscle content directly influences CW. That making the phenotypic correlation between REA and CW the highest among the phenotypic correlations between CW and other characteristics.

Mortimer et al [10] reported genetic parameter estimates within a single-breed analysis, including heritability estimates for lean meat yield, loin muscle weight, topside weight, weight of fat trim from the loin, and hind leg bone weight, where direct heritability ranged from 0.17 to 0.46. Van Heelsum et al [28] estimated the heritability of lean meat percentage to be 0.46. Earlier studies of animals have described the heritability range for lean meat percentage in specific areas, such as the shoulder, leg, or loin, to be between 0.27 and 0.37 [29–32]. Some studies, like this one, used computed tomography to study carcass composition in carcasses and estimated the heritability range for different carcass traits to be between 0.2 and 0.5 [33–36]. In our results, heritability estimates for carcass fat were generally lower than those for carcass lean. However, the phenotypic variability in carcass fat was much greater, suggesting that selection for altered fat levels may lead to greater variation compared to selection for carcass lean. This is highly advantageous for population improvement.

We estimated the heritability of REA (0.37) and BF (0.50), with similar results observed across species. Heritability for traits associated with REA ranged from 0.19 to 0.44, and for BF, it ranged from 0.28 to 0.64 [10,37–40]. REA and CFP show a strong positive genetic correlation, while CMP shows a negative genetic correlation. In contrast, BF shows the opposite correlation, indicating that REA and BF are genetically linked to carcass composition traits. Research reports on GWAS have identified common genetic loci for these traits [41]. This connection is particularly evident in the genetic correlation results for MTB and CBP, which showed the highest genetic correlation in this study. Matika et al [29] conducted a GWAS analysis on Scottish Blackface lambs and found that the significant region of MTB overlapped with a subregion of the significant region of CBP. Due to polygenic effects or linkage disequilibrium (LD), strong genetic correlations can arise between certain traits. While this may be beneficial for breeding processes, it can also lead to antagonistic effects between traits. Further investigation is needed to explain the genetic link in more detail.

Genomic evaluation is widely used for genetic selection in various farm animals worldwide [42], particularly for carcass meat quality traits, where its role is well-established. We estimated the genetic selection accuracy for carcass traits and found that the selection accuracy for carcass component traits ranged from 0.34 to 0.43. In pigs and cattle, genetic selection accuracy for carcass traits ranged from 0.33 to 0.72 [43,44]. Our results were relatively lower compared to those in pigs and cattle, primarily due to the smaller reference population size [45]. Brito et al [46] estimated the GS accuracy for traits such as REA, BFT, and CW using a population of 14,845 sheep, with accuracy ranging from 0.14 to 0.33. The estimated range of accuracy in this study is from 0.29 to 0.43. Although the sample size in this study is relatively small, the results are close to those of Brito et al’s research. The populations in this study were constructed under similar age and nutritional conditions. In addition, previous researchers used the ultrasonic method for measurement in their studies, and there were relatively large human errors in the measurement process, which were not as highly consistent as the measurement standard procedures in this study. Therefore, despite its small size, the reference population still maintains a high level of quality.

When conducting genomic selection, the selection of high-quality breeding stock plays a significant role in the genetic progress of offspring. We evaluated the genetic progress resulting from direct selection of carcass traits based on selection intensity, as well as the selection response to CMP and CFP after selecting for carcass traits. Since CFP and CMP traits are difficult to measure, constructing a genomic selection evaluation process is one of the most effective ways to improve these traits. If constructing a direct genomic selection reference population is not feasible, improving CMP and CFP through strong genetic correlations is also an effective approach. For indirect selection, a relative efficiency close to 100% can achieve the same results as direct selection. We found that selecting REA and BF can effectively improve both CFP and CMP based on GEBV, providing a foundational guide for breeding sheep with enhanced CMP and CFP.

## CONCLUSION

The consistency and quality carcasses have previously been identified as a significant barrier to the expansion of the sheep. This study presents genetic parameter estimates for carcass traits in terminal sire breeds population and provides the necessary parameters for their genetic evaluation, and estimated Genomic selection accuracy utilizing the ssGBLUP approach. Carcass traits were found to be moderately to strongly heritable, indicating a substantial potential for improving carcass yield and quality through genetic selection. Slaughter traits that are positively genetically correlated with carcass composition traits may serve as useful predictors in live animals, which would be useful for breeding desirable traits for lean meat production ability and meat quality. When large-scale CT phenotyping is not possible, carcass quality can be improved by selecting REA vs. BF for indirect selection of CMP and CFP.

## Figures and Tables

**Table 1:** Descriptive analysis of carcass traits in mutton sheep

Trait	Unit	Mean	SD	Max	Min	CV
Carcass fat percentage (CFP)	%	23.33	5.92	43.04	10.03	25.120
Carcass muscle percentage (CMP)	%	64.03	5.71	77.03	45.05	8.863
Carcass bone percentage (CBP)	%	12.74	1.15	16.01	10.01	8.943
Meat-to-bone ratio (MTB)	-	6.962	0.697	9.000	5.188	10.008
Carcass weight (CW)	kg	28.078	4.223	43.810	16.880	15.040
Loin eye area (REA)	mm^2^	1,966.615	285.094	3,171.600	1,175.500	14.497
Backfat thickness (BF)	mm	5.017	1.646	12.100	2.100	32.818
GR	cm	9.261	2.460	20.400	2.600	26.568
Dressing percentage (DP)	%	53.06	2.95	67.06	34.02	5.401

CV = SD/Mean×100%.

GR: the total tissue depth of muscle and fat at the twelfth rib, 110 mm from the midline MTB is a ratio of two traits and thus has no unit.

SD, standard deviation; Max, maximum; Min, minimum; CV, coefficient of variation.

**Table 2:** Variance components and heritability estimates for carcass traits in mutton sheep

Trait	$\sigma_{a}^{2}$	se	$\sigma_{e}^{2}$	se	*h*^2^±se
CFP	15.11	5.25	17.4	4.39	0.47±0.15
CMP	15.20	4.98	14.5	4.09	0.51±0.15
CBP	0.44	0.17	0.72	0.14	0.38±0.13
MTB	0.17	0.06	0.27	0.05	0.39±0.14
CW	5.47	2.37	4.70	1.98	0.54±0.21
REA	28,581.6	12,394.8	48,527.5	10,708.1	0.37±0.15
BF	1.23	0.41	1.24	0.34	0.5±0.15
GR	0.81	0.59	4.65	0.60	0.15±0.11
DP	1.07	0.85	6.51	0.84	0.14±0.11

$\sigma_{a}^{2}=\text{additive variance}$; se = standard error; $\sigma_{e}^{2}=\text{residual variance}$; *h*^2^ = the heritability for each trait.

CFP, carcass fat percentage; CMP, carcass muscle percentage; CBP, carcass bone percentage; MTB, meat-to-bone ratio; CW, carcass weight; REA, loin eye area; BF, backfat thickness; DP, dressing percentage.

**Table 3:** Direct and correlated genetic responses per generation and relative efficiency (%) for CFP and CMP under selection for carcass traits

Trait	Unit	Direct response	Correlated response		Relative efficiency of selection (%)	
	
			CFP	CMP	CFP	CMP
CFP	%	2.30	-	−2.26	-	99.47
CMP	%	2.25	−2.20	-	104.27	-
CBP	%	0.36	−0.71	0.31	322.60	727.27
MTB	-	0.19	0.58	−0.24	396.56	929.09
CW	kg	0.93	0.72	−0.53	317.44	420.99
REA	mm^2^	88.23	−1.79	1.70	128.30	132.50
BF	mm	0.64	1.69	−1.75	135.74	128.35
GR	cm	0.45	1.05	−0.77	219.51	293.77
DP	%	0.53	−0.81	0.90	283.39	250.43

Direct and indirect genetic progression were estimated based on a selection intensity of 1.2. Direct response refers to the genetic progression of each trait when selected directly. Correlated response refers to the genetic progression of CMP and CFP when selected for other traits. Direct response and indirect response units are both original trait units. The relative efficiency of selection is compared to the genetic progression following direct selection for CMP and CFP with the genetic progression of CMP and CFP resulting from the selection of other traits.

CFP, carcass fat percentage; CMP, carcass muscle percentage; CBP, carcass bone percentage; MTB, meat-to-bone ratio; CW, carcass weight; REA, loin eye area; BF, backfat thickness; DP, dressing percentage.

## Data Availability

Upon reasonable request, the datasets of this study can be available from the corresponding author.

## References

[1] Yu TY Morton JD Clerens S Dyer JM In-depth characterisation of the lamb meat proteome from longissimus lumborum EuPA Open Proteom 2015 6 28 41 10.1016/j.euprot.2015.01.001 PMC4510072 26217735

[2] Luo Z Ou H McSweeney CS Tan Z Jiao J Enhancing nutrient efficiency through optimizing protein levels in lambs: involvement of gastrointestinal microbiota Anim Nutr 2025 20 332 41 10.1016/j.aninu.2024.09.006 40034464 PMC11872659

[3] Daza A Rey AI Lopez-Carrasco C Lopez-Bote CJ Influence of feeding system on growth performance, carcass characteristics and meat and fat quality of Avileña-Negra Ibérica calves’ breed Spanh J Agric Res 2014 12 409 18 10.5424/sjar/2014122-4096

[4] Tian Y Zhao Y Yao Y Genetic and functional validation of CTSS in regulating intramuscular fat content of Duroc–Landrace–Yorkshire pigs Anim Genet 2025 56 e70010 10.1111/age.70010 40251967

[5] Arikawa LM Mota LFM Schmidt PI Genome-wide scans identify biological and metabolic pathways regulating carcass and meat quality traits in beef cattle Meat Sci 2024 209 109402 10.1016/j.meatsci.2023.109402 38056170

[6] Lopez BIM Song C Seo K Genetic parameters and trends for production traits and their relationship with litter traits in Landrace and Yorkshire pigs Anim Sci J 2018 89 1381 8 10.1111/asj.13090 30073716

[7] Gozalo-Marcilla M Buntjer J Johnsson M Genetic architecture and major genes for backfat thickness in pig lines of diverse genetic backgrounds Genet Sel Evol 2021 53 76 10.1186/s12711-021-00671-w 34551713 PMC8459476

[8] Lee SH Kim S Kim JM Genetic correlation between biopsied and post-mortem muscle fibre characteristics and meat quality traits in swine Meat Sci 2022 186 108735 10.1016/j.meatsci.2022.108735 35065370

[9] Mortimer SI Fogarty NM van der Werf JHJ Genetic correlations between meat quality traits and growth and carcass traits in Merino sheep J Anim Sci 2018 96 3582 98 10.1093/jas/sky232 29893862 PMC6127828

[10] Mortimer SI Hatcher S Fogarty NM Genetic correlations between wool traits and carcass traits in Merino sheep J Anim Sci 2017 95 2385 98 10.2527/jas.2017.1385 28727038

[11] Ding R Zhuang Z Qiu Y Identify known and novel candidate genes associated with backfat thickness in Duroc pigs by large-scale genome-wide association analysis J Anim Sci 2022 100 skac012 10.1093/jas/skac012 35034121 PMC8867564

[12] Kodama Y Shumway M Leinonen R The sequence read archive: explosive growth of sequencing data Nucleic Acids Res 2012 40 D54 6 10.1093/nar/gkr854 22009675 PMC3245110

[13] Bolger AM Lohse M Usadel B Trimmomatic: a flexible trimmer for Illumina sequence data Bioinformatics 2014 30 2114 20 10.1093/bioinformatics/btu170 24695404 PMC4103590

[14] Li H Durbin R Fast and accurate short read alignment with Burrows–Wheeler transform Bioinformatics 2009 25 1754 60 10.1093/bioinformatics/btp324 19451168 PMC2705234

[15] McKenna A Hanna M Banks E The genome analysis toolkit: a MapReduce framework for analyzing next-generation DNA sequencing data Genome Res 2010 20 1297 303 10.1101/gr.107524.110 20644199 PMC2928508

[16] Danecek P Auton A Abecasis G The variant call format and VCFtools Bioinformatics 2011 27 2156 8 10.1093/bioinformatics/btr330 21653522 PMC3137218

[17] Cingolani P Platts A Wang LL A program for annotating and predicting the effects of single nucleotide polymorphisms, SnpEff: SNPs in the genome of Drosophila melanogaster strain w1118; iso-2; iso-3 Fly 2012 6 80 92 10.4161/fly.19695 22728672 PMC3679285

[18] Yin L Zhang H Tang Z HIBLUP: an integration of statistical models on the BLUP framework for efficient genetic evaluation using big genomic data Nucleic Acids Res 2023 51 3501 12 10.1093/nar/gkad074 36809800 PMC10164590

[19] Johnson DL Thompson R Restricted maximum likelihood estimation of variance components for univariate animal models using sparse matrix techniques and average information J Dairy Sci 1995 78 449 56 10.3168/jds.S0022-0302(95)76654-1

[20] Falconer DS Introduction to quantitative genetics Pearson Education India 1996

[21] Moraes GF Abreu LRA Toral FLB Selection for feed efficiency does not change the selection for growth and carcass traits in Nellore cattle J Anim Breed Genet 2019 136 464 73 10.1111/jbg.12423 31328836

[22] Kava R Peripolli E Brunes LC Estimates of genetic and phenotypic parameters for feeding behaviour and feed efficiency-related traits in Nelore cattle J Anim Breed Genet 2023 140 264 75 10.1111/jbg.12756 36633154

[23] Cavanagh CR Jonas E Hobbs M Thomson PC Tammen I Raadsma HW Mapping quantitative trait loci (QTL) in sheep. III. QTL for carcass composition traits derived from CT scans and aligned with a meta-assembly for sheep and cattle carcass QTL Genet Sel Evol 2010 42 36 10.1186/1297-9686-42-36 20846385 PMC2949606

[24] Davoli R Catillo G Serra A Genetic parameters of backfat fatty acids and carcass traits in large white pigs Animal 2019 13 924 32 10.1017/S1751731118002082 30152309

[25] Zhang F Wang Y Mukiibi R Genetic architecture of quantitative traits in beef cattle revealed by genome wide association studies of imputed whole genome sequence variants: I: feed efficiency and component traits BMC Genomics 2020 21 36 10.1186/s12864-019-6362-1 31931702 PMC6956504

[26] Zuin RG Buzanskas ME Caetano SL Genetic analysis on growth and carcass traits in Nelore cattle Meat Sci 2012 91 352 7 10.1016/j.meatsci.2012.02.018 22405874

[27] McGilchrist P Alston CL Gardner GE Thomson KL Pethick DW Beef carcasses with larger eye muscle areas, lower ossification scores and improved nutrition have a lower incidence of dark cutting Meat Sci 2012 92 474 80 10.1016/j.meatsci.2012.05.014 22717222

[28] van Heelsum AM Lewis RM Davies MH Haresign W Genetic relationships among objectively and subjectively assessed traits measured on crossbred (mule) lambs Anim Sci 2006 82 141 9 10.1079/ASC200523

[29] Matika O Riggio V Anselme-Moizan M Genome-wide association reveals QTL for growth, bone and in vivo carcass traits as assessed by computed tomography in Scottish blackface lambs Genet Sel Evol 2016 48 1 15 10.1186/s12711-016-0191-3 26856324 PMC4745175

[30] Conington J Bishop SC Waterhouse A Simm G A comparison of growth and carcass traits in Scottish blackface lambs sired by genetically lean or fat rams Anim Sci 1998 67 299 309 10.1017/S1357729800010067

[31] Johnson PL Lopez-Villalobos N Bryant JR Blair HT Genetic parameters for carcass cuts using data from a progeny test of Romney rams Proceedings of the 8th World Congress on Genetics Applied to Livestock Production 2006 Aug 13–18 Belo Horizonte, Brazil CABI 2006

[32] Lorentzen TK Vangen O Genetic and phenotypic analysis of meat quality traits in lamb and correlations to carcass composition Livest Sci 2012 143 201 9 10.1016/j.livsci.2011.09.016

[33] Jones HE Lewis RM Young MJ Simm G Genetic parameters for carcass composition and muscularity in sheep measured by X-ray computer tomography, ultrasound and dissection Livest Prod Sci 2004 90 167 79 10.1016/j.livprodsci.2004.04.004

[34] Karamichou E Richardson RI Nute GR McLean KA Bishop SC Genetic analyses of carcass composition, as assessed by X-ray computer tomography, and meat quality traits in Scottish blackface sheep Anim Sci 2006 82 151 62 10.1079/ASC200518

[35] Kvame T Vangen O Selection for lean weight based on ultrasound and CT in a meat line of sheep Livest Sci 2007 106 232 42 10.1016/j.livsci.2006.08.007

[36] Lambe NR Conington J Bishop SC Relationships between lamb carcass quality traits measured by X-ray computed tomography and current UK hill sheep breeding goals Animal 2008 2 36 43 10.1017/S1751731107001061 22444961

[37] Barro AG Marestone BS dos SER Genetic parameters for frame size and carcass traits in Nellore cattle Trop Anim Health Prod 2023 55 71 10.1007/s11250-023-03464-z 36757607

[38] Keele JW Foraker BA Boldt R Kemp C Kuehn LA Woerner DR Genetic parameters for carcass traits of progeny of beef bulls mated to dairy cows J Anim Sci 2024 102 skae075 10.1093/jas/skae075 38489760 PMC10989647

[39] Arikawa LM Mota LFM Schmidt PI Genetic parameter estimates for carcass and meat quality traits and their genetic associations with sexual precocity indicator traits in Nellore cattle J Anim Breed Genet 2025 142 581 93 10.1111/jbg.12927 39907255

[40] Pauling RC Speidel SE Thomas MG Holt TN Enns RM Genetic parameters for pulmonary arterial pressure, yearling performance, and carcass ultrasound traits in Angus cattle J Anim Sci 2023 101 skad288 10.1093/jas/skad288 37698445 PMC10563144

[41] Xie L Qin J Rao L Genetic dissection and genomic prediction for pork cuts and carcass morphology traits in pig J Anim Sci Biotechnol 2023 14 116 10.1186/s40104-023-00914-4 37660101 PMC10475202

[42] Walsh JB Genomic selection signatures and animal breeding J Anim Breed Genet 2021 138 1 3 10.1111/jbg.12527 33314373

[43] Iqbal A Choi TJ Kim YS Comparison of genomic predictions for carcass and reproduction traits in Berkshire, Duroc and Yorkshire populations in Korea Asian-Australas J Anim Sci 2019 32 1657 63 10.5713/ajas.18.0672 31480201 PMC6817783

[44] Lopes FB Baldi F Brunes LC Genomic prediction for meat and carcass traits in Nellore cattle using a Markov blanket algorithm J Anim Breed Genet 2023 140 1 12 10.1111/jbg.12740 36239216

[45] Misztal I Aguilar I Lourenco D Ma L Steibel JP Toro M Emerging issues in genomic selection J Anim Sci 2021 99 skab092 10.1093/jas/skab092 33773494 PMC8186541

[46] Brito LF Clarke SM McEwan JC Prediction of genomic breeding values for growth, carcass and meat quality traits in a multi-breed sheep population using a HD SNP chip BMC Genetics 2017 18 7 10.1186/s12863-017-0476-8 PMC5267438 28122512

